# Regulation of Adipocyte Differentiation by METTL4, a 6 mA Methylase

**DOI:** 10.1038/s41598-020-64873-w

**Published:** 2020-05-19

**Authors:** Zhenxi Zhang, Yingzi Hou, Yao Wang, Tao Gao, Ziyue Ma, Ying Yang, Pei Zhang, Fan Yi, Jun Zhan, Hongquan Zhang, Quan Du

**Affiliations:** 1 State Key Laboratory of Natural and Biomimetic Drugs, School of Pharmaceutical Sciences, Peking University, Beijing, 100191 China; 2 Department of Stomatology, Beijing Friendship Hospital, Capital Medical University, 95 Yong’an Road, Western District, Beijing, 100050 China; 3 Key Laboratory of Carcinogenesis and Translational Research (Ministry of Education), Peking University Health Science Center, Beijing, 100191 China

**Keywords:** DNA, DNA, Differentiation, Differentiation, DNA methylation

## Abstract

As one of the most abundant DNA methylation form in prokaryotes, N^6^-methyladenine nucleotide (6 mA) was however only recently identified in eukaryotic genomes. To explore the implications of N^6^-adenine methylation in adipogenesis, genomic N^6^-adenine methylation was examined across adipocyte differentiation stages of 3T3-L1 cells. When the N^6^-adenine methylation profiles were analyzed and compared with the levels of gene expression, a positive correlation between the density of promoter 6 mA and gene expression level was uncovered. By means of *in vitro* methylation and gene knockdown assay, METTL4, a homologue of *Drosophila* methylase CG14906 and *C. elegans* methylase DAMT-1, was demonstrated to be a mammalian N^6^-adenine methylase that functions in adipogenesis. Knockdown of *Mettl4* led to altered adipocyte differentiation, shown by defective gene regulation and impaired lipid production. We also found that the effects of N^6^-adenine methylation on lipid production involved the regulation of INSR signaling pathway, which promotes glucose up-taking and lipid production in the terminal differentiation stage.

## Introduction

As a major epigenetic modification, genomic DNA methylation is of critical importance to many cellular processes such as expressional regulation, imprinting, X chromosome inactivation, and tumorigenesis^1,2^. Until very recently, 5-methylcytosine (5mC) has been known to be the only DNA methylation form in eukaryotic genomes^3^. This is however different from that of prokaryotes, in which 6 mA is another major DNA methylation form and involved in expressional regulation, genome replication as well as restriction-modification systems^4,5^. The presence of 6 mA was recently revealed in diverse eukaryotic genomes, including *Chlamydomonas reinhardtii, Caenorhabiditis elegans, Drosophila melanogaster*, zebrafish, pig, *Arabidopsis thaliana*, mouse and humans^6–13^. ALKBH1 was identified in 2016 as a 6 mA demethylase involved in the regulation of long interspersed nuclear element1 (LINE-1)^13^. More recently, 6 mA was found to be involved in tumorigenesis, and its effects were mediated by ALKBH1 and N6AMT1. N6AMT1 was reported in 2018 to be the first mammalian 6 mA methylase^14^. Despite those progresses, many essential aspects of the metabolism and biological consequences of 6 mA remain to be known.

Adipocyte differentiation of 3T3-L1 preadipocytes has been used as a classic *in vitro* model system of adipogenesis. Induced by an MDI cocktail consisting of insulin, dexamethasone and 3-isobutyl-1-methyxanthine, the preadipocytes exit from cell proliferation and enter into terminal differentiation stage, to generate matured adipocytes. During this process, the cells undergo stages of growth-arrest’, hormone induction, mitotic clonal expansion, and terminal differentiation. Widespread epigenetic modifications have been recently revealed during the differentiation, shown by dynamic chromosome modifications and complex gene regulation^15^. Genomic 5mC methylations have been found to be involved in this process, regulating the expression of many adipogenic factors such as *Fabp4*, *Glut4* and *Lep*^16–18^. However, whether N^6^-adenine methylation is also involved in the MDI-induced maturation of adipocytes is still unknown. In the present study, we examined the genomic distribution of 6 mA by means of immunoprecipitation/sequencing assay (6mA-IP-seq), and explored its roles in adipocyte differentiation of 3T3-L1 cells and the underlying mechanisms.

## Results

### Dynamic changes of genomic N^6^-adenine methylation during adipocyte differentiation

To explore the involvement of N^6^-adenine methylation in adipogenesis, the extents of genome-wide modification were determined during the differentiation of 3T3-L1 cells. Upon induction with MDI, proliferating 3T3-L1 cells undergo the process of differentiation, which recapitulates the major events occurring *in vivo*. To facilitate the description of the process, the day to perform MDI induction is called day(0). Accordingly, the cells grew to confluence on day(−2) and entered into the “growth-arrest” stage from day(−2). After MDI induction on day(0), the cells proceeded to a mitotic clonal expansion stage and entered into a terminal differentiation stage from day(+2) on.

**Figure 1:**
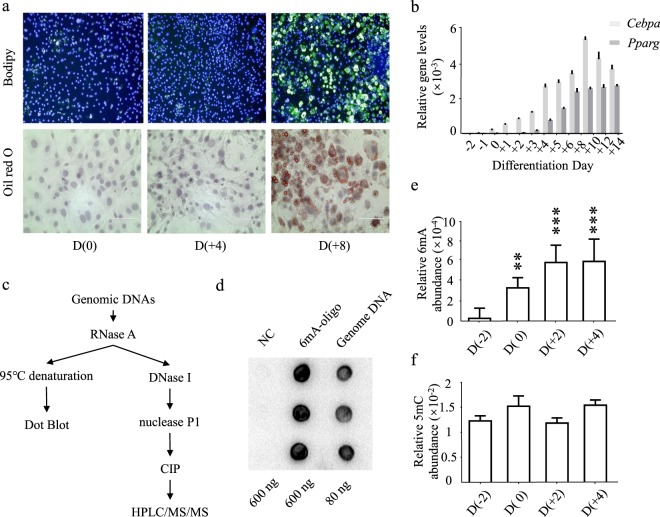
Profiling of N^6^-adenine methylation in adipocyte differentiation of 3T3-L1 cells. (**a**) Staining of cellular lipid of preadipocytes on Day(0), differentiating adipocytes on Day(+4), and mature adipocytes on Day(+8). Lipid staining was performed with Bodipy 493/503 (Green) and Oil red O (Red), nuclei were co-stained with Hoechst 33342 (Blue). (**b**) Expression profiles of *Cebpa* and *Pparg* were determined by qRT-PCR, in relative to the expression of *β-actin*. (**c**) Workflow to quantify 6 mA abundance of genome DNA sample. (**d**) Dot blot assay of N^6^-adenine methylation. NC, a DNA oligo without N^6^-adenine methylation. 6mA-oligo, a DNA oligo containing one N^6^-adenine methylation. Genome DNA, genome DNAs extracted from 3T3-L1 preadipocytes. (**e,f**) Abundances of 6 mA and 5mC methylation were quantified using HPLC-MS/MS analysis. Molar ratios of 6 mA to dA were calculated, and molar ratios of 5mC to dC were calculated. Data are presented as mean ± s.d, n > 3 independent assay. *P < 0.05; **P < 0.01; ***P < 0.001.

To monitor the progress of differentiation, Bodipy and Oil red O staining were performed (Fig. 1a). In addition to checking the production of cellular lipids, regulation of the major adipogenic factors were also examined by gene expression assay (Fig. 1b). By means of dot blot assay, the presence of genomic N^6^-adenine methylation was confirmed in proliferating 3T3-L1 cells (Fig. 1c,d). To rule out potential contamination of bacteria or mycoplasma, assays were performed with universal 16 S primer sets (Supplementary Fig. S1a)^10^, and a commercial mycoplasma detection kit (Supplementary Fig. S1b).

To quantitatively determine the presence of 6 mA, genomic DNAs were extracted from the cells collected on day(−2) before MDI treatment and day(0), day(+2) and day(+4) after MDI treatment, representing proliferating preadipocytes, growth-arresting cells, differentiating adipocytes, and matured adipocytes. DNA samples were then sequentially degraded with RNase, DNase I and nuclease P1. The resulting nucleoside products were separated and quantified using a LC-MS/MS system^19^, with dA, dC, 5mC and 6 mA as the standards. The abundance of genomic 6 mA was presented as the molar ratio to dA. In proliferating preadipocytes, a relatively low level (0.01%) of 6 mA was found. With the progress of differentiation, 6 mA levels gradually increased and reached a peak of 0.06% at the terminal stage (Fig. 1e). Comparing with 6 mA, 5mC showed a relatively stable profile during the process (Fig. 1f).

### Genomic N^6^-adenine methylation detected by immunoprecipitation and sequencing

Genomic N^6^-adenine methylation was further investigated by immunoprecipitation and sequencing (6mA-IP-seq). 6mA-IP-seq was performed with DNA samples of preadipocytes and matured adipocytes. The genomic DNAs were sonicated into fragments of 100–500 bp, ligated to adaptors with unique sequence index, denatured to single-stranded DNA, and immuno-precipitated to obtain fragments carrying N^6^-adenine methylation. 6mA-containing fragments were PCR amplified, and sequenced on an Illumina HiSeq. 2000 platform. From a total of 118 million sequencing reads, 114 million clean reads were obtained. This led to a clean read ratio of 0.9689, much higher than the threshold ratio of 0.75 that indicated a high-quality sequencing data. Sequencing reads were then mapped to mouse reference genome (NCBI37/mm10), using TopHat algorithm. By means of a peak-finding algorithm^20^, 6 mA sites were individually identified. This led to the identification of ~10,000 high-confidence 6 mA sites in preadipocytes and mature adipocytes, with FDR < 0.01. Furthermore, 6 mA immunoprecipitation/qPCR (6mA-IP-qPCR) was performed to confirm the methylation status of 7 randomly selected positive Peaks and 2 negative Peaks (Supplementary Fig. S2).

### Genome-wide distribution of N^6^-adenine methylation

**Figure 2:**
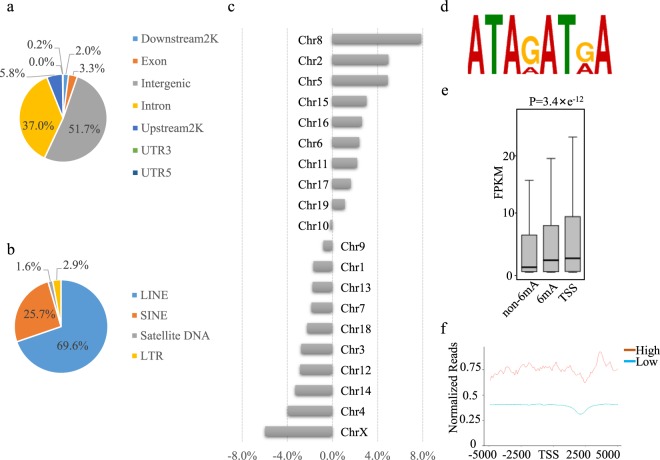
6mA-IP-seq and RNA-seq of mature adipocytes. (**a**) Genome-wide distribution of N^6^-adenine methylation. (**b**) Distribution profiles of 6 mA in transposon elements. (**c**) Chromosome-wide distribution of 6 mA. For each chromosome, a chromosome length ratio was calculated by dividing its length in nucleotide against the total length of the genome, a 6 mA ratio was calculated by dividing the number of its 6 mA sites against the number of the total 6 mA sites. Then, an 6 mA abundance index was calculated by subtracting the 6 mA ratio by its chromosome length ratio. (**d**) Sequence motifs of 6 mA sites. (**e**) Comparison of the median and interquartile range of gene expression between genes without 6 mA sites (Non-6mA), genes with 6 mA sites (6 mA) and genes with 6 mA sites around TSS region (TSS), in 3T3-L1 cell of Day(+4). (**f**) 6 mA profile around TSS region, for genes with high (FPKM > 50) and low (FPKM < 50) expression level. Reads densities were normalized by RPKM algorithm.

To understand the genome-wide distribution of 6 mA, locus-specific enrichment of 6 mA sites was examined in terms of gene exons, introns, 5’-UTR, 3’-UTR, upstream2K regions (within 2 kb upstream of TSS), downstream 2 K regions (within 2 kb downstream of TES), and intergenic regions. In mature adipocyte cells, 51.7% of the methylations were found within intergenic region, much higher than the background ratio calculated from the input (Fig. 2a). Our results were consistent with the reported profiles for mouse brain and embryonic stem cells^13,21^. For the 6 mA sites in intergenic regions, 69.6% was found within long interspersed nuclear elements (LINEs). However, no locus-specific preference was observed to distribute within the major TEs, including short interspersed nuclear elements (LINEs), long terminal repeated (LTR) and satellite DNA (Fig. 2b).

Further examination found that 6 mA sites were enriched on chromosome 8, 2 and 5 (Fig. 2c), which was different from that found in embryonic stem cells where enrichment was found on chromosome X^13^. On the other hand, similar 6 mA distribution profiles were found for preadipocytes and mature adipocytes (Supplementary Fig. S3a–c).

Among the 6 mA sites identified, ATARATRA(R = A or G) was shown to be the most enriched motif using DREME software, with an E-value of 1.0e^−038^ (Fig. 2d). This motif shares the sequence of AAATA with another 6 mA motif GRAATA identified in mouse ESCs^13^.

### A positive correlation between 6 mA abundance and gene expression

To examine the implications of N^6^-adenine methylation in differentiation, further analyses were performed with gene expression data obtained by RNA-seq. Based on the presence of 6 mA sites in a genomic region, protein-coding genes were divided into 6mA-containing group and 6mA-absent group. As shown in Fig. 2e, the median and interquartile FPKM (fragments per kilobase of transcript per million mapped reads) values of the 6mA-containing group were much higher than those of the 6mA-absent group. This result demonstrated that the expression levels of 6mA-containing genes were much higher than that of 6mA-absent genes. When the analyses were performed with genes carrying at least one 6 mA site at their TSS (400-bp window centered on the TSS), this correlation was much more remarkable.

To confirm the observation in another way, genes were divided into high expression (FPKM > 50) and low expression groups (FPKM < 50). The abundances of 6 mA sites in these two groups were analyzed and compared. As expected, genes of the high expression group displayed higher 6 mA abundance at TSS regions, comparing with the genes of low expression (Fig. 2f, Supplementary Fig. S3d). Taken together, a positive correlation was concluded between N^6^-adenine methylation of a gene and its expression.

### Characterization of a mammalian N^6^-adenine methylase

Potential 6 mA metabolic enzymes were investigated subsequently. To this aspect, DNA 6 mA methylases and demethylases characterized in the other species are summarized in Supplementary Fig. S4. Mammalian homologues of these enzymes were searched by querying Uniprot database. In addition to N6AMT1 that was characterized as the first mammalian N^6^-adenine methylase^12^, METTL4 was identified as a candidate of N^6^-adenine methylase, due to its high similarity to *Drosophila* methylase CG14906 and *C. elegans* methylase DAMT-1. Additionally, TETs and ALKBH4 were found homologous to *Drosophila* demethylase DMAD and *C. elegans* demethylase NMAD.

**Figure 3:**
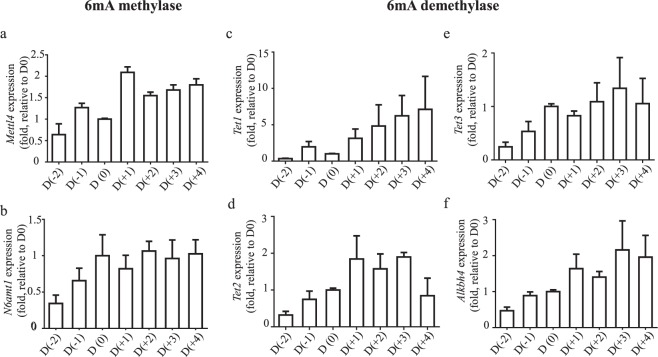
Expression regulation of 6 mA methylase and demethylase. During adipocyte differentiation of 3T3-L1 cells, gene expression of *Mettl4* (**a**)*, N6amt1* (**b**)*, Tet1* (**c**)*, Tet2* (**d**)*, Tet3* (**e**) and *Alkbh4* (**f**) were determined by quantitative RT-PCR and presented.

**Figure 4:**
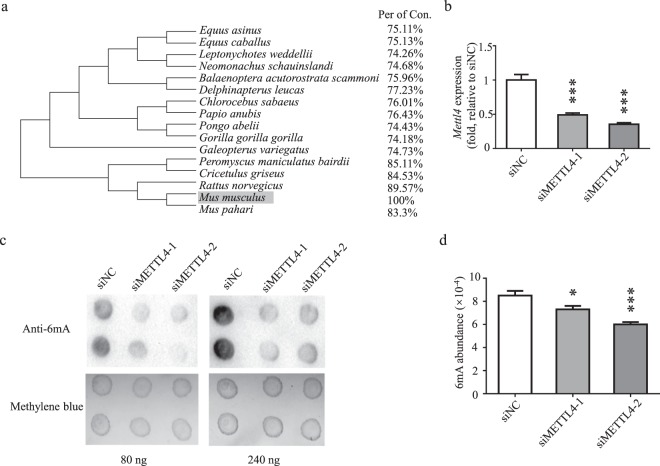
Knock-down of *Mettl4* leads to decreased 6 mA levels. (**a**) Phylogenetic tree analysis of METTL4 in mammals. (**b**) 3T3-L1 cells were transfected with *Mettl4*-targeting siRNAs. With the cells, gene expression of *Mettl4* was determined by quantitative RT-PCR, in relative to control cells treated by a sequence-irrelevant siRNA. (**c,d**) Genomic 6 mA levels were determined by dot blot (**c**) and HPLC-MS/MS. Full-length blots are presented in Supplementary Fig. S5 (**d**). Data are analyzed by Student’s t-test and presented as mean ± s.d., n = 3 independent experiments. *P < 0.05; **P < 0.01; ***P < 0.001.

To examine their involvement in adipocyte differentiation, expression profiling was performed for *Mettl4*, *N6amt1*, *Tets* and *Alkbh4*. Similar expression profiles were found for *Mettl4* and *N6amt1* (Fig. 3), which were consistent with the increased formation of 6 mA in the genomic DNA. Phylogenetic tree analysis indicated that METTL4 protein is highly conserved in mammals (Fig. 4a).

To examine the role of *Mettl4* in N^6^-adenine methylation, gene knockdown assay was carried out with *Mettl4*-targeting siRNAs (Fig. 4b). Two days after siRNA transfection, genomic DNAs were extracted from siRNA-treated and untreated cells. The abundances of 6 mA were examined by dot blot assay. It was shown that knock-down of *Mettl4* led to significantly decreased 6 mA levels (Fig. 4c, Supplementary Fig. S5a,b). Consistently, HPLC-MS/MS assay found that the abundance of 6 mA decreased by ~30% in *Mettl4-*knockdown cells (Fig. 4d).

### Catalytic activity of METTL4 *in vitro*

To assess the catalytic activity of METTL4, *in vitro* methylation assays were performed with recombinant METTL4 protein, according to the reported methylation procedures^22^. Recombinant METTL4 protein was produced using a prokaryotic expression system^23^. *In vitro* methylation assays were performed with two single-stranded oligo substrates, and the products were analyzed by dot blot assay and HPLC-MS/MS analysis.

**Figure 5:**
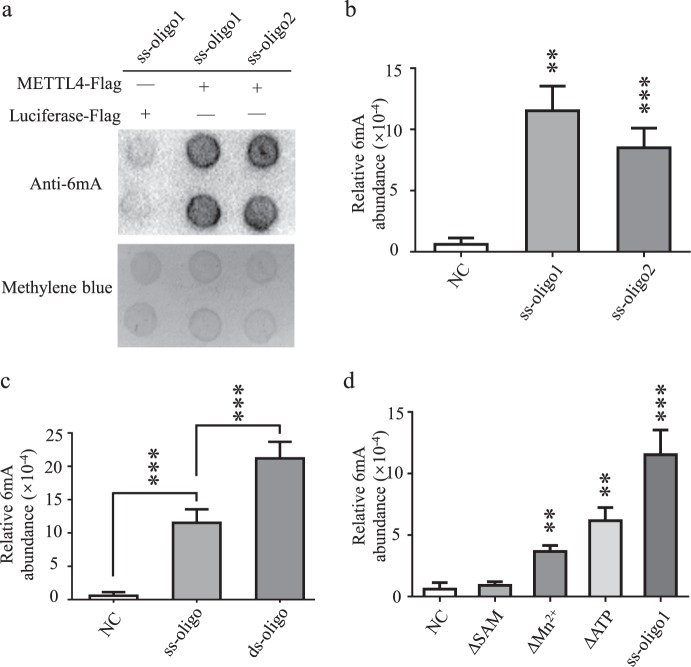
Catalytic activity of METTL4 *in vitro*. (**a,b**) Catalytic activity of N^6^-adenine methylation of recombinant METTL4. Two single-stranded DNA oligos were used as reaction substrates for recombinant METTL4-Flag protein. An irrelevant *Firefly*-luciferase was included as a negative control. DNA substrates were subjected to dot blotting assay using an anti-6mA antibody (**a**) and LC-MS/MS assay (**b**). For dot blotting assay, 600 ng of DNA was loaded for each group and full-length blots are presented in Supplementary Fig. S6. (**c**) Methylation efficacies on single-stranded (ss-oligo) and double-stranded (ds-oligo) substrates. (**d**) Optimization of methylation reaction. Data are analyzed by Student’s t-test and presented as mean ± s.d., n = 3 independent experiments. **P < 0.01; ***P < 0.001.

Dot blot assay indicated the formation of 6 mA modified products (Fig. 5a,b, Supplementary Fig. S6a,b). HPLC-MS/MS analysis found that about 0.1% of the adenine nucleotides were converted to 6 mA after incubated with 0.2 μg METTL4 for 12 h. The conversion efficiency was comparable to that of N6AMT-1^12^.

To explore the substrate preference of METTL4, methylation was performed with single- or double-stranded oligo substrates. Results showed that the methylation efficiency of double-stranded substrates was about twice as high as that of single-stranded substrates (Fig. 5c). Moreover, the effects of methyl donor SAM, ATP and Mn^2+^ on the methylation reaction were examined (Fig. 5d). Among these factors, SAM was found to be the most critical factor, exhibiting the greatest influence on N^6^-adenine methylation. The second most critical factor was Mn^2+^, and the absence of Mn^2+^ led to decreased catalytic activity by about 70%. The absence of ATP decreased the catalytic activity by about 50%.

These *in vivo* and *in vitro* investigations demonstrated that METTL4 was a second mammalian N^6^-adenine methylase, in addition to the firstly reported N6AMT-1^12^.

### The effects on adipocyte differentiation

**Figure 6:**
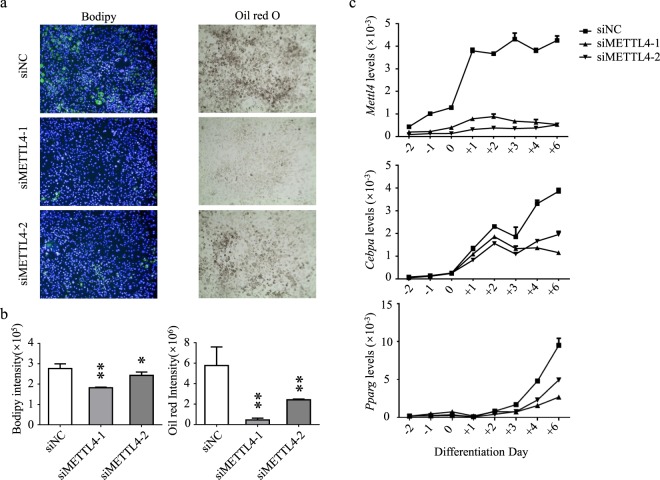
Knockdown of *Mettl4* leads to altered adipocyte differentiation. (**a**) The effects of *Mettl4* knockdown were monitored by Bodipy staining on day(+4) (Left panel, 10×), and Oil Red O staining on day(+8) (Right panel, 10×). (**b**) Quantitative analyses of Bodipy (Left panel) and Oil Red O staining (Right panel). (**c**) The effects on gene expression of the major adipogenic factors. Data are analyzed by Student’s t-test and presented as mean ± s.d., n = 3 independent experiments. **P < 0.01; ***P < 0.001.

To examine the roles of *METTL4* in adipocyte differentiation, gene knockdown assays were performed. 3T3-L1 cells were firstly transfected with gene-specific siRNA on day(−2), treated with MDI cocktail on day(0), transfected again with gene-specific siRNA on day(+1), and harvested on day(+4) or day(+8). Bodipy and Oil Red O staining were performed to check the progress of differentiation. Comparing with control cells, *Mettl4* knock-down cells showed greatly altered differentiation and decreased lipid production as much as 70% (Figs. 6a,b, Supplementary Fig. S7).

The effects on adipocyte differentiation were also confirmed by altered major adipogenic factors. Consistent with defective differentiation phenotype, expressions of *Pparg* and *Cebpa* in *Mettl4* knock-down cells decreased to 41% and 30% of the normal levels (Fig. 6c).

### N^6^-adenine methylation in the gene promoters by METTL4

We hypothesized that METTL4 exerts its action by increasing N^6^-adenine methylation in the promoters of downstream adipogenic genes during adipocyte differentiation, which increases their expression and facilitates the progress of differentiation. On the contrary, knockdown of *Mettl4* decreases promoter methylation of the genes, resulting in decreased gene expression and altered cell differentiation.

**Figure 7:**
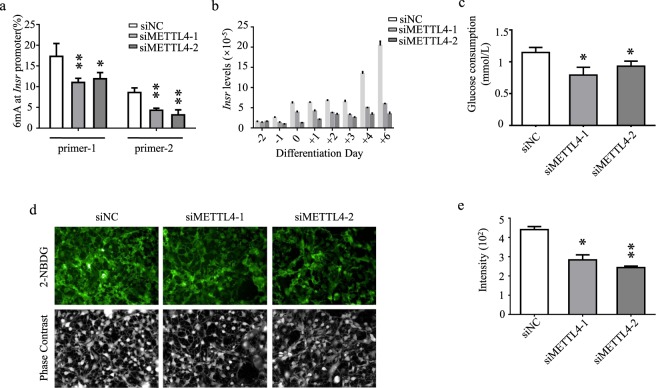
The effects of *Mettl4* knockdown on glucose metabolism. (**a**) Promoter 6 mA abundance of *Insr* gene on day(+2) of the differentiation was determined by 6mA-IP-qPCR. (**b**) Expressional profiles of *Insr* in wild-type and *Mettl4* knockdown cells. Gene expression levels were determined by quantitative RT-qPCR, in relative to that of β-actin. (**c**) Knockdown of *Mettl4* led to decreased consumption of glucose. (**d**) Fluorescence images of 2-NBDG uptake. (**e**) Quantitative analyses of 2-NBDG uptake. Data are analyzed by Student’s t-test and presented as mean ± s.d., n = 3 independent experiments. *P < 0.05; **P < 0.01.

To confirm this hypothesis, we examined the N^6^-adenine methylation status of a group of adipogenic factors, including *CEBPα*, *CEBPβ*, *PPARγ*, *WNT4*, *INSR*, *FAS*, and *PDE-3B* in wild-type and *Mettl4* knocked-down cells. Genomic DNAs were extracted on day(+2) and day(+4). For each of the genes, promoter 6 mA levels were determined using 6mA-IP-qPCR. Anti-IgG antibody was included in the assay as a negative control, and at least two independent primer sets were checked for each 6 mA site (Supplementary Fig. S8a). Results showed that promoter methylation was decreased by 30–50% for *Insr* gene in *Mettl4* knockdown cells (Fig. 7a, Supplementary Fig. S9a). This was in contrast with the relatively stable levels of 6 mA in the promoters of *Cebpa, Cebpb, Pparg, Wnt4, Fas* and *Pde-3b* (Supplementary Figs. 8b–g, 9b–g). When gene expression was examined, a decrease of 65% was found with *Insr* expression (Fig. 7b).

### The effects of METTL4 on glucose metabolism

We proposed that INSR signaling pathway is involved in the process. In adipocyte differentiation, INSR pathway is involved in GLUT4 translocation from transport vesicles to cell membrane, therefore promoting transmembrane absorption of glucose. When the translocation of GLUT4 is repressed, both glucose absorption and consumption will be affected, resulting in decreased lipid production^24,25^.

To investigate the roles of METTL4 in glucose metabolism, glucose consumption (GC) assays were performed with wild-type and *Mettl4* knock-down cells. On day(+4) of differentiation, culture medium was replaced with DMEM containing 1000 mg/L glucose and 0.2% BSA. The glucose levels were examined daily by means of glucose oxidase assay^26^. Comparing with control cells treated with a sequence-irrelevant siRNA, knockdown of *Mettl4* led to decreased glucose consumption by 20–30% (Fig. 7c).

To investigate the roles of METTL4 in glucose uptake, glucose absorption assay was also performed^27^. On day(+4) of differentiation, an equal amount of 2-NBDG, a glucose transport probe, was added to each well of wild-type and *Mettl4* knockdown cells. After incubation for 30 minutes, cellular distribution of 2-NBDG was examined. It was shown that knockdown of *Mettl4* resulted in significantly reduced up-take of glucose probe by the cells (Fig. 7d). Comparing with control cells, fluorescence intensity decreased by about 50% in *Mettl4* knockdown cells (Fig. 7e). Therefore, *Mettl4* knockdown led to decreased glucose up-taking and consumption, decreased lipid production and altered adipocyte differentiation.

## Discussion

DNA methylation plays critical roles in many biological processes including differentiation of adipocytes. In this study, we investigated the roles of N^6^-adenine methylation in the differentiation of 3T3-L1 preadipocytes. Comparing with the relatively stable levels of 5mC, dynamic changes of N^6^-adenine methylation were found, suggesting its involvement in the differentiation process of adipocytes. Genomic-wide distribution of 6 mA was examined by 6mA-IP-seq and 51.7% of the N^6^-adenine methylation sites was found within the intergenic regions. Chromosomes 2 and 8 were found to be the most enriched ones, accounting for 12.6% and 11.6% of the total N^6^-adenine methylation. In contrast, chrX was accounted for only 0.3% of the methylation sites. Compared with the 6 mA profile reported for mouse embryonic stem cells^13^, cell-type-specific distribution patterns were revealed. For ESCs, 74.7% of the 6 mA sites were distributed within the intergenic regions, chromosome X was the most 6mA-enriched chromosome and chromosome 8 was the least enriched chromosome.

A positive correlation between gene expression and the levels of N^6^-adenine methylation, in particularly at the promoter regions, was found. Highly expressed genes were found to be associated with increased promoter N^6^-adenine methylation, while genes of low expression were found to be associated with decreased 6 mA levels. Although the levels of 6 mA were found positively correlated with gene expression in general, locus- or sequence-specific roles needs to be further elucidated, considering the added effects of genomic 5mC methylation. 5mC methylations are usually found within CpG islands of promoter regions, functioning in gene inactivation^28^. However, when the methylations were located in coding region of a gene, an opposite effect on gene transcription was reported^29^.

During the differentiation of 3T3-L1 cells, the abundance of genome 6 mA was found to increase gradually. METTL4, a homologue of *Drosophila* methylase CG14906 and *C. elegans* methylase DAMT-1, was demonstrated to be a mammalian N^6^-adenine methylase. Knockdown of *Mettl4* not only led to decreased 6 mA levels, but also altered the differentiation process of 3T3-L1 cells. We found that knockdown of *Mettl4* led to decreased N^6^-adenine methylation at the promoter region of *Insr* gene, down-regulated gene expression and inactivated the INSR pathway. With the progress of adipocyte differentiation, expression of 6 mA methyltransferase *Mettl4* starts to increase. This leads to up-regulation of promoter methylation and expression of downstream genes, including *Insr*. In the case of *Insr* gene, up-regulation of its expression activates INSR signaling pathway, increases glucose up-taking of the cells, and promotes lipid production of adipocytes. In summary, we identified the second mammalian N^6^-adenine methylase METTL4 and showed N^6^-adenine methylation played critical roles in the differentiation of adipocytes.

Taken together, these data indicated that INSR pathway is involved in the process. However, learning from the study on 5mC methylation, activities of 6 mA are speculated to be complex and diverse. In the differentiation of 3T3-L1 cells, very likely INSR pathway is one of the mechanisms affected by N^6^-adenine methylation. The effects on the major adipogenic regulators, such as PPARγ and CEBPβ, might contribute to the process to a larger extent.

Besides *Mettl4*, gene expression profiling found that a similar profile was also shared by *N6amt1*, another 6 mA methylase characterized in mammalian. Active regulation was also revealed for the other potential 6mAenzymes examined, likely suggests their involvement in the process. However, to the effects on methylation of specific gene, a complicated DNA methylation process will be implicated. Furthermore, different modification enzymes likely have their distinguishable downstream genes, which process may be guided by a variety of lncRNAs^30^.

## Methods

### Cell culture and adipocyte differentiation

3T3-L1 preadipocytes were maintained in Dulbecco’s Modified Eagle’s Medium (DMEM, Thermo Scientific) supplemented with 10% calf serum (Invitrogen) at 37 °C and under 5% CO_2_ air. Two days after reaching confluence, cells were induced to differentiation by replacing the medium with DMEM containing 10% fetal bovine serum (Invitrogen), 500 μM isobutyl-methylxanthine (Sigma), 1 μM dexamethasone (MP) and 1 μg/mL insulin (Invitrogen). Cells were then incubated for 6–8 days with medium replacement every 2 days^31^.

### BODIPY and Oil Red O staining

For BODIPY staining, cells were fixed in 4% paraformaldehyde for 15 min, permeabilized in PBS containing 1% Triton X-100 for 15 min, and stained with PBS containing BODIPY 493/503 (Invitrogen) and Hoechst 33342 (M&C) for 45 min at 37 °C. Analyses were performed with a High Content Analysis (Operetta).

For Oil Red O staining, cells were fixed with 70% ethanol for 10 min, stained with Oil Red O (Solarbio) in isopropanol for 30 min, and washed three times with 70% ethanol. For quantitative measurements of lipid accumulation, cells were washed with PBS to remove excess of stain solution, dried, and dissolved in 100% isopropanol. Absorbance was measured spectrophotometrically with FlexStation 3 Multi-Mode Microplate Reader (Molecular Devices) at 520 nm^31^.

### Gene silencing assay

Cells were plated in complete growth medium with 10% serum. siRNAs (Ribobio) and Lipofectamine RNAiMAX reagent (Invitrogen) were added to serum-free medium, and incubated at room temperature for 15 min. The transfection complex mixture was then added to the cells and incubated for 24–48 hr before analysis^31^.

### RNA isolation and RT-qPCR

RNAs were isolated using TRIzol reagent (Invitrogen). cDNAs were synthesized using HiscriptII Q RT SuperMix for qPCR kit (Vazyme, Nanjing). qPCR was performed using GoTaq qPCR Master Mix (Promega). Data were normalized to the expression of β-actin gene^31^.

### RNA-seq

RNAs were collected from preadipocyte cells on Day(−2) and matured adipocytes Day(+4) and the amount and quality of RNAs were determined by NanoPhotometer®(IMPLEN, CA, USA), Qubit®3.0 Flurometer (Life Technologies, CA, USA) and 2100 RNA Nano 6000 Assay Kit (Agilent Technologies, CA, USA)^10^. RNA-seq assays were performed on an Illumina Hiseq 2500 platform and clean reads were mapped to mouse genome (GRCm38/mm10) using HISAT2 v2.1.0^32^. Value of FPKM was calculated to represent the expression level of genes by HTSeq (http://www-huber.embl.de/users/anders/HTSeq/doc/overview.html)^33^.

### Dot Blot assay

Genomic DNAs were isolated using TIANamp Genomic DNA Kit (TIANGEN) and diluted to a concentration of 80 ng/μL. DNA samples were heated at 95 °C for 10 min to denature DNA, placed immediately on ice for 5 min, loaded at an amount of 120 ng or 240 ng per dot on a Hybond+ membrane. Membranes were air dried, irradiated by UV for 2 min to auto-crosslink DNA, blocked for 1 hr in 5% milk TBST, and probed with primary antibody (1:2000, Synaptic Systems) in 5% milk TBST at 4 °C overnight. After washing for five times with TBST, the membranes were probed with secondary antibody in 5% milk for 3 hr at room temperature, and washed for five times with TBST. Hybridization signals were detected with an Immobilon Western Chemilum HRP Substrate (Merck Millipore)^7^.

### Enzymatic hydrolysis of genomic DNA

Genomic DNAs were isolated and treated first by RNase A for 12 hr at 50 °C, purified using TIANquick Maxi Purification Kit (TIANGEN). Purified DNA of 1 μg was sequentially treated by DNase I (8 U) for 12 hr at 37 °C, Nuclease P1 (8 U) for 12 hr at 50 °C, and calf intestinal alkaline phosphatase (1 U) for 12 hr at 37 °C. The hydrolysis products were dried in a vacuum centrifugal concentrator and washed by acetonitrile for 2 times. The resulting nucleoside-containing fractions were reconstituted in ultrapure water to a final concentration of 2 mg/mL, before HPLC-MS/MS assay^19^.

### HPLC-MS/MS assay

Nucleosides sample of 10 μL was added to a 10 μL 6-Cl-Purine solution (3 mg/mL) and filtered through a 0.22 mm filter. 5 μL of the solution was subjected to LC-Ion Trap assay. DNA methylation was analyzed with a LC-ESI-MS/MS system consisting of a Shimadzu LC-20A HPLC system (Shimadzu, Kyoto, Japan) and an ABSciex QTRAP 5500 (AB Sciex, Canada). Data acquisition and processing were performed using AB SCIEX Analyst 1.5.2 Software (Applied Biosystems, CA). LC separation was performed using an Atlantis T3 column (4.6 mm * 150 mm, 5 mm, Waters) (WatersCorp., MA, USA) with a flow rate of 0.3 mL/min at 40 °C. 3 mM ammonium formate (solvent A) and methanol (solvent B) were used as mobile phases. A gradient of 2 min 2% B, 4 min 2–5% B, 2 min 5–10% B, 4 min 10% B, 0.1 min 10–95% B, 2 min 95% B, 0.1 min95–2% B, 4.9 min 2% B was used. Positive electrospray ionization mode was used to perform the mass spectrometry detection. Target analytes were monitored by multiple reaction monitoring (MRM) mode using the mass transitions (precursor ions/productions) of dC (228.1/112.1), dT (243.2/127.1), dA (252.2/136.0), dG (268.1/152.1), 5mC (242.0/126.0), 6 mA (266.1/150.1), 6-Chloropurine (6-CIP, 287.0/155.0). 6-CIP was spiked in as the internal standard in all the assays, due to its optimal detection sensitivity. Using MRM mode, the assays revealed a lineal relationship for tested nucleosides, with a lineal range of 10–10000 ng/mL for dA, 20–1000 ng/mL for dC, 2–1000 ng/mL for 5mC, and 0.1–100 ng/mL for 6 mA. The coefficients of correlation were determined to be higher than 0.98. The MRM parameters of all nucleosides were optimized to achieve maximal detection sensitivity. Quantification was performed by comparing with standard curves. A relative ratio was calculated for each nucleoside, based on the calculated molar concentrations^19^.

### 6mA-IP-sequencing

Using a Bioruptor, genomic DNAs were sonicated to fragments of 200–500 bp. DNA adaptors were then ligated to DNA fragments following the Illumina protocol. After denaturing at 95 °C for 5 mins, the resulted single-stranded DNA fragments were immunoprecipitated with 6 mA antibodies (Synaptic Systems) at 4 °C overnight. Together with input DNAs, 6mA-enriched DNA fragments were purified according to Active Motif hMeDIP protocol, PCR amplified using Illumina indexing primers, subjected to library construction and sequencing using an Illumina HiSeq 2000. The experiments, including 6 mA IP, library construction and sequencing, were performed at Annoroad Gene Technology(Beijing, China). Low-quality reads were removed from raw data by using the Trimmomatic package (http://www.usadellab.org/cms/uploads/supplementary/Trimmomatic/Trimmomatic-Src-0.35.zip) and clean data were aligned to the mouse genome (UCSC, mm10), using Bowtie2. Peaks calling of 6 mA were performed by means of Macs2 software^20^ and the p-value was set as<e^−5 32^.

### 6mA-IP-qPCR

Using a Vibra-Cell Ultrasonic Liquid Processor (Sonics & Materials Inc, Newtown, USA), genomic DNA was sheared to an average of 200–1000 bp and denatured. 1 mg of denatured DNA was incubated with 1 mg anti-6mA antibody (Synaptic Systems) in IP buffer (1 mM sodium phosphate buffer pH 7.0, 0.14 M NaCl, 0.05% Triton X-100) for 3 hr at 4 °C. Antibody-bound DNA was collected with 10 μL of anti-mouse IgG dynabeads (Invitrogen) overnight at 4 °C with a rotating wheel, washed three times with IP buffer, recovered in 200 μL digestion buffer (50 mM Tris-HCl pH 8.0, 10 mM EDTA pH 8.0, 0.5% SDS, 40 μg proteinase K), and incubated at 56 °C for 2 hr with occasional mixing by vortexing. Recovered DNA was purified and quantified by qPCR assay. The ratio of genes in 6mA-IP group to input was calculated to reach the 6 mA abundance in specific locus. Sequences of the primers for gene detection are included as Supplementary Information^31^.

### Cloning and Expression of METTL4

Full-length coding sequence of mouse METTL4 (GenBank: NP_001344064.1) was synthesized and subcloned into a pMCSG19 vector, generating a plasmid named pMCSG19-His-METTL4. METTL4 proteins were expressed in BL21 (DE3) *E. coli* strain, and purified for activity characterization.

### Glucose consumption assay

3T3-L1 cells were grown in DMEM (4500 mg/L glucose) containing 10% fetal bovine serum and were plated into 96-well plates. Two days after confluence, the cells were introduced to differentiation, and transfected with siRNA on day(−2) and day(+1) for two times. Glucose consumption assays were performed by replacing culture medium with DMEM containing 1000 mg/L glucose and 0.2% BSA. After the assay, culture medium was collected and the glucose concentration was determined by the glucose oxidase method. Comparing with the glucose concentrations of blank wells, glucose consumption (GC) of plated wells was calculated.

### Glucose uptake assay

Cells were plated into 6-well plates and induced to differentiation two days after reaching confluence. Four days after induction, culture medium was removed from the culture wells, the cells were washed with PBS for 3 times, and fresh PBS containing 2-NBDG of1mmol/L was added. The cell plate was incubated for 30 min at 37 °C. The cells were washed for 3 times before High Content Analysis using Operetta.

### *In vitro* methylation assay

A methylation reaction of 25 μL contained 10 mM HEPES (pH7.9), 10 mM MgCl_2_, 160 mM KCl, 5 mM SAM, 20 μM ATP, 0.02 μg protein and 2 ng DNA oligo. Assays were performed by incubating at 37 °C for 12 hours, and stopped by heating to 95 °C for 5 min. Sequences of DNA oligos are included in the Supplementary Information.

### Statistical analysis

Analyses were performed with GraphPad Prism5 software. Data was presented as mean ± SD. ANOVA and Student’s t-test were performed to evaluate the statistical significance. The significance level was set at p < 0.05.

## Supplementary information

Supplementary information.

## Data Availability

The data of 6mA-seq and RNA-seq had been uploaded to SRA database and the accession was PRJNA588576.
