# Supplementary material for: Regulation of Adipocyte Differentiation by METTL4, a 6 mA Methylase
Source: Sci Rep. 2020 May 19;10:8285. doi: 10.1038/s41598-020-64873-w (PMC7237444; doi:10.1038/s41598-020-64873-w)
Supplement: Supplementary file 1 — Supplementary information. [file 41598_2020_64873_MOESM1_ESM.pdf]

Regulation of Adipocyte Differentiation by METTL4, a 6mA Methylase

Zhenxi Zhang[1], Yingzi Hou[1], Yao Wang[1], Tao Gao[1], Ziyue Ma[1], Ying Yang[2], Pei Zhang[1], Fan Yi[1], Jun Zhan[1,3], Hongquan Zhang[1,3], Quan Du[1*]

[1] State Key Laboratory of Natural and Biomimetic Drugs, School of Pharmaceutical Sciences, Peking University, Beijing, 100191, China.

[2] Department of stomatology, Beijing Friendship Hospital, Capital Medical University, NO. 95 Yong'an Road, Western District, Beijing, 100050, China.

[3] Key Laboratory of Carcinogenesis and Translational Research (Ministry of Education), Peking University Health Science Center, Beijing 100191, China.

[*] Correspondence should be addressed to Dr. Quan Du, Tel: +86-010-82805780, Email: quan.du@pku.edu.cn.

Supplemental Figure S1.

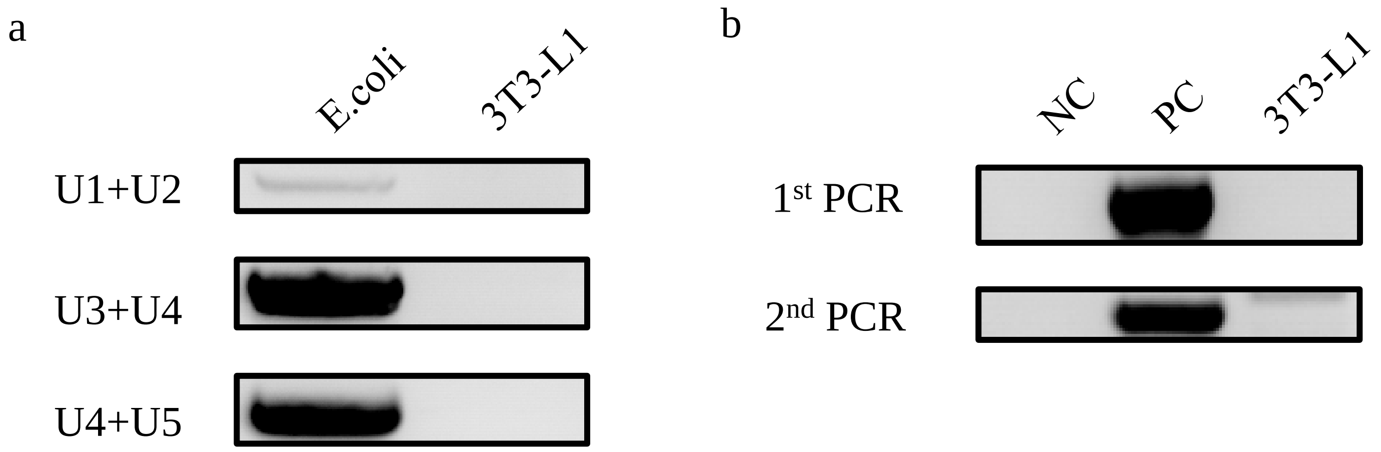

Supplemental Figure S1. Experiments to rule out potential contamination.

(a) PCR assays were performed with universal 16S primers, to exclude possible bacterial contamination. (b) Commercial mycoplasma detection kits (40611ES25, YEASEN) were used to exclude possible contamination of mycoplasma.

Supplemental Figure S2.

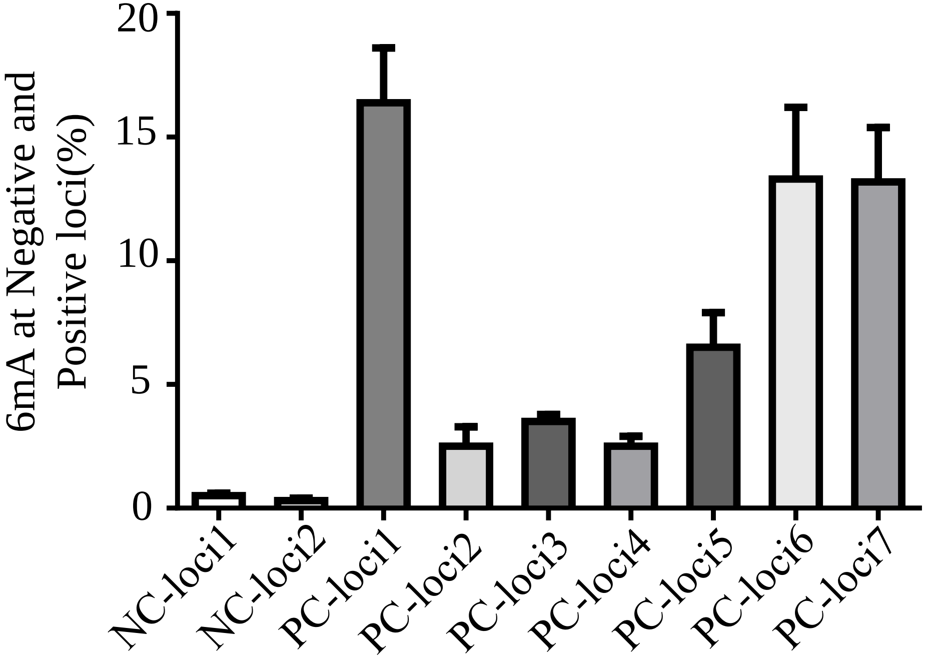

Supplemental Figure S2. Validation of 6mA methylation by 6mA-IP-qPCR.

6mA methylation were evaluated by 6mA-IP-qPCR at negative-loci and positive-loci.

Supplemental Figure S3.

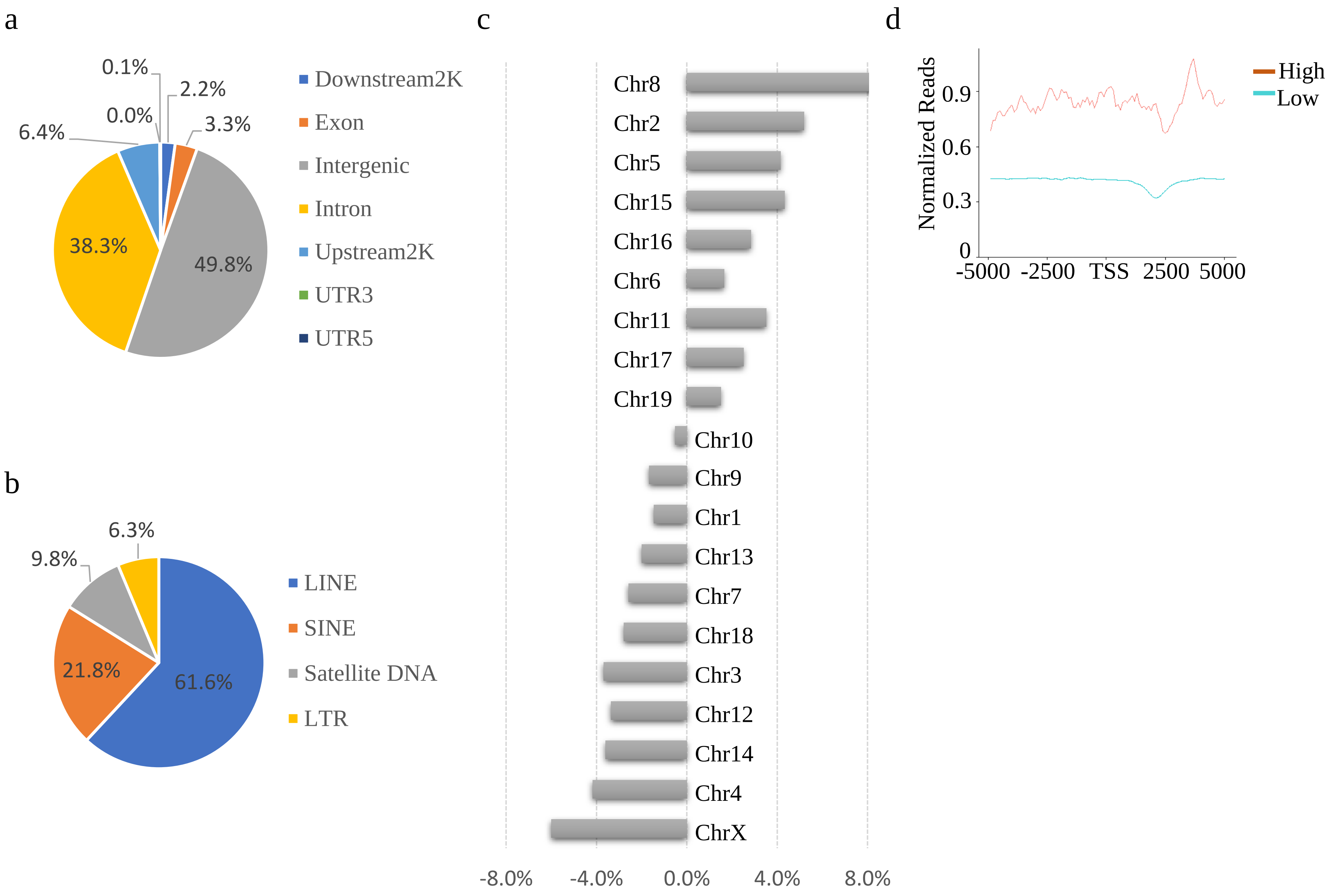

Supplemental Figure S3. 6mA-IP-seq and RNA-seq of preadipocytes. (a) Genome-wide distribution profile of 6mA methylation. (b) Distribution profile of 6mA in transposon elements. (c) Chromosome-wide distribution of 6mA. For each chromosome, a chromosome length ratio was calculated by dividing its length in nucleotide against the total length of the genome, a 6mA ratio was calculated by dividing the number of its 6mA sites against the number of the total 6mA sites. Then, an 6mA abundance index was calculated by subtracting its 6mA ratio by its chromosome length ratio. (d) 6mA profile around TSS region, for genes with high (FPKM >50) and low (FPKM <50) expression level. Reads densities were normalized by RPKM algorithm. p values were calculated by a two-tailed unpaired Student’s t test.

Supplemental Figure S4.

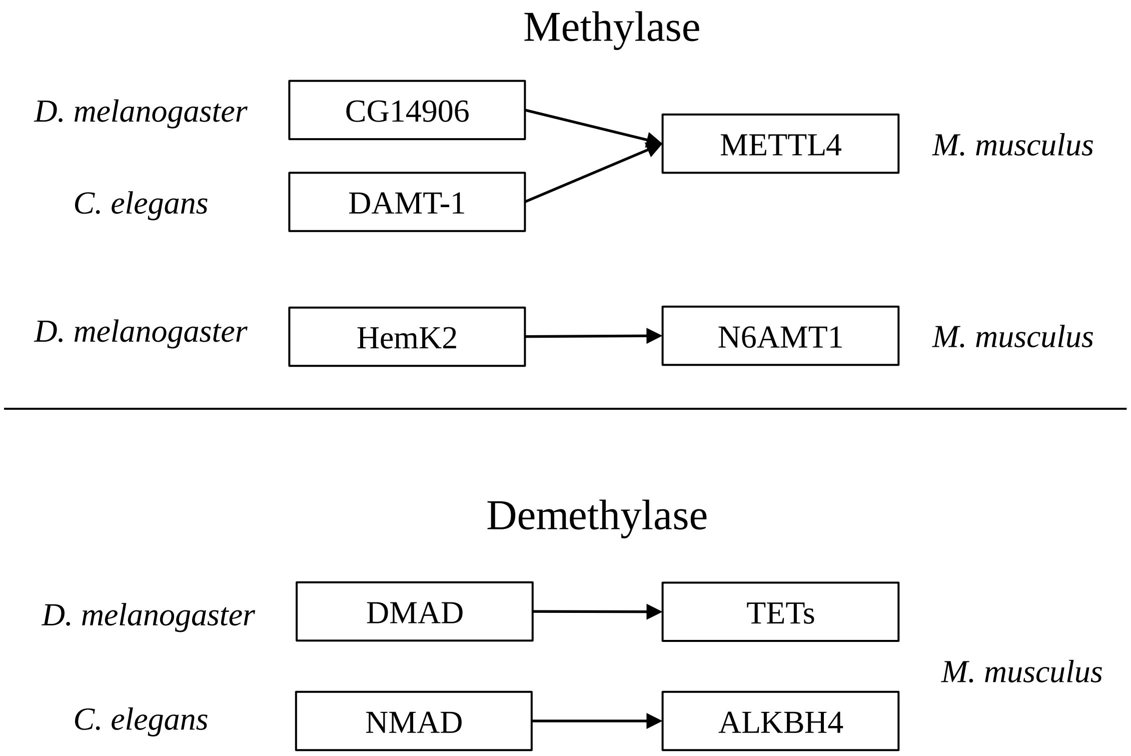

Supplemental Figure S4. Summary of DNA 6mA methylases and demethylases.

6mA methylases and demethylases characterized in the other eukaryotic organisms were summarized, together with their potential mammalian homologous proteins.

Supplemental Figure S5.

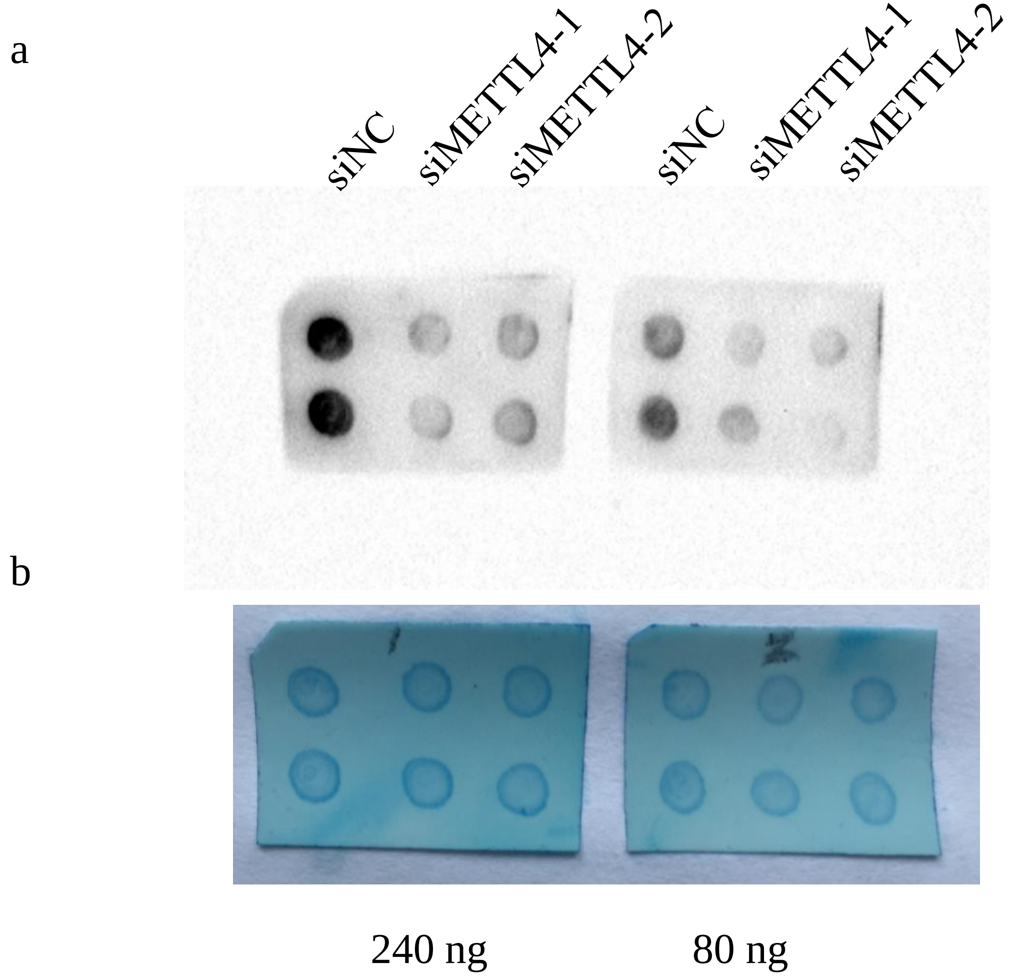

Supplemental Figure S5. Original images of figure 4c. (a) Dot Blot for Genomic 6mA levels. (b) Methylene blue for loaded DNA staining.

Supplemental Figure S6.

a

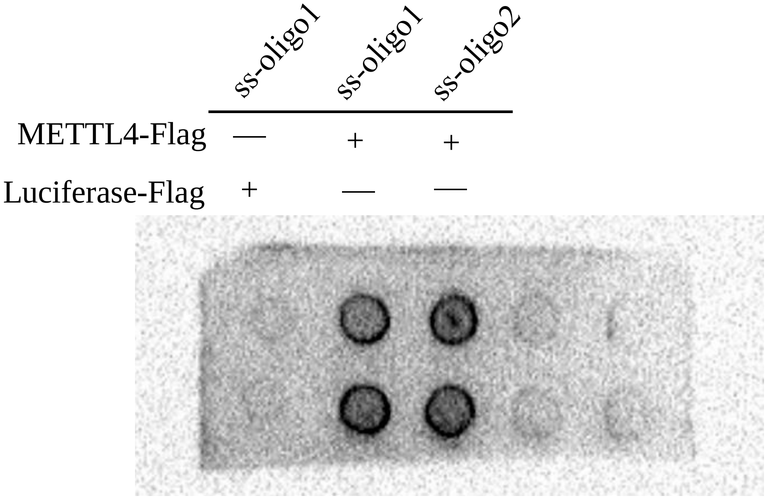

b

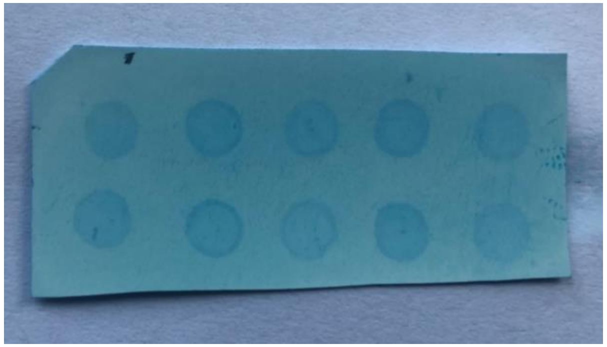

Supplemental Figure S6. Original images of figure 5a. (a) Dot Blot for DNA oligos in methylation assays. (b) Methylene blue for loaded DNA staining.

Supplemental Figure S7.

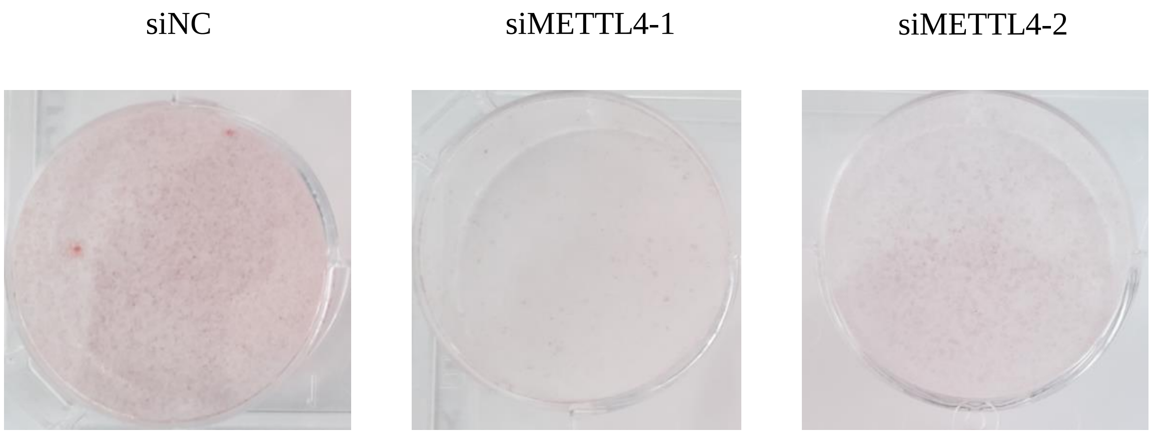

Supplemental Figure S7. Images of oil red O staining. Images of oil red O staining were presented in the format of a whole incubation well, to show the repression effects of *Mettl4* knockdown.

Supplemental Figure S8.

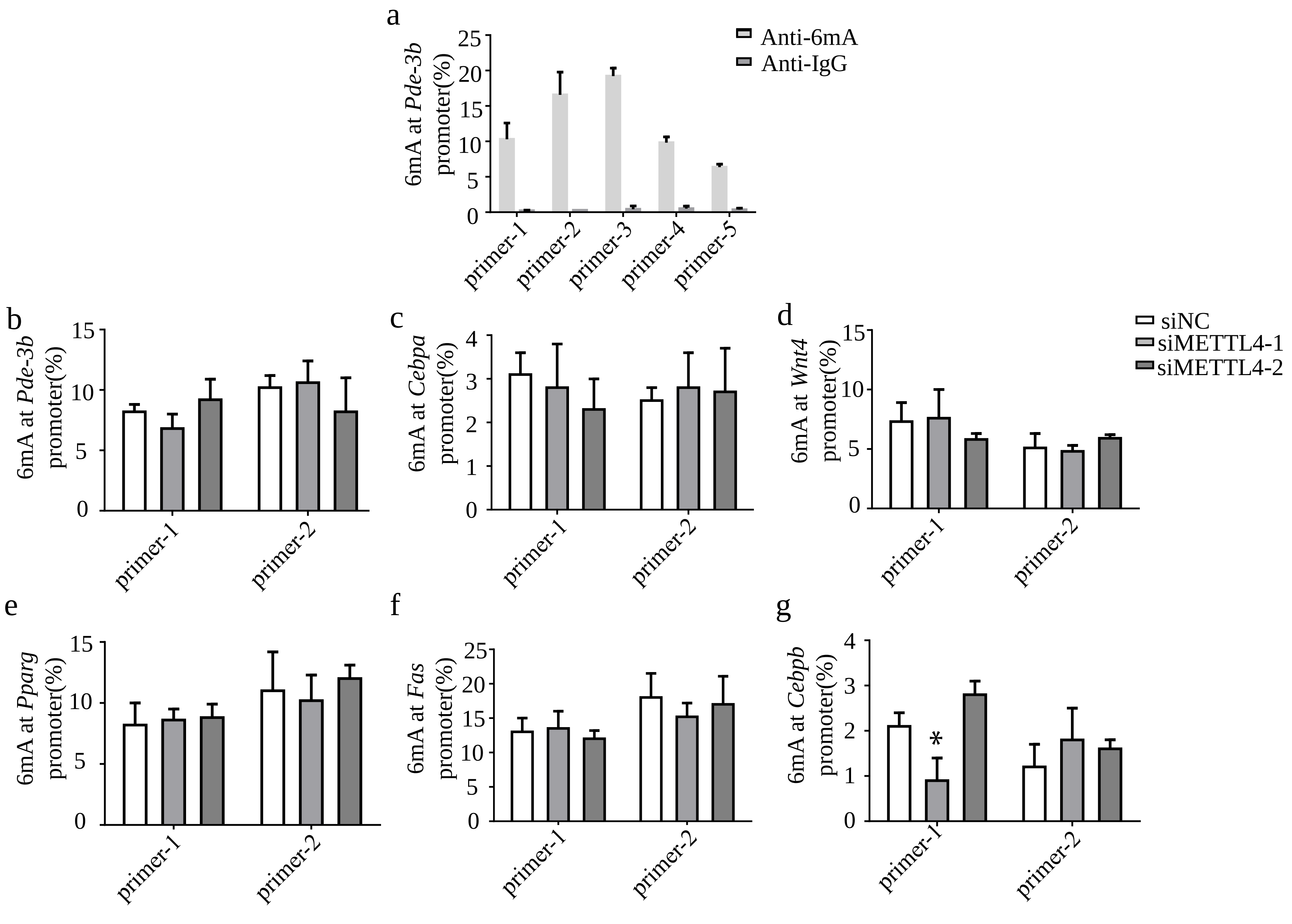

Supplemental Figure S8. Effects of *Mettl4* knockdown on day(+2) of the differentiation. 6mA-IP-qPCR was performed to examine the effects of *Mettl4* knockdown on promoter methylation of *Pde-3b, Cebpa, Wnt4, Pparg, Fas and Cebpb*. (a) Validation of the assay, anti-IgG antibody was used as a negative control. (b-g) Promoter 6mA methylation of the genes.

Supplemental Figure S9.

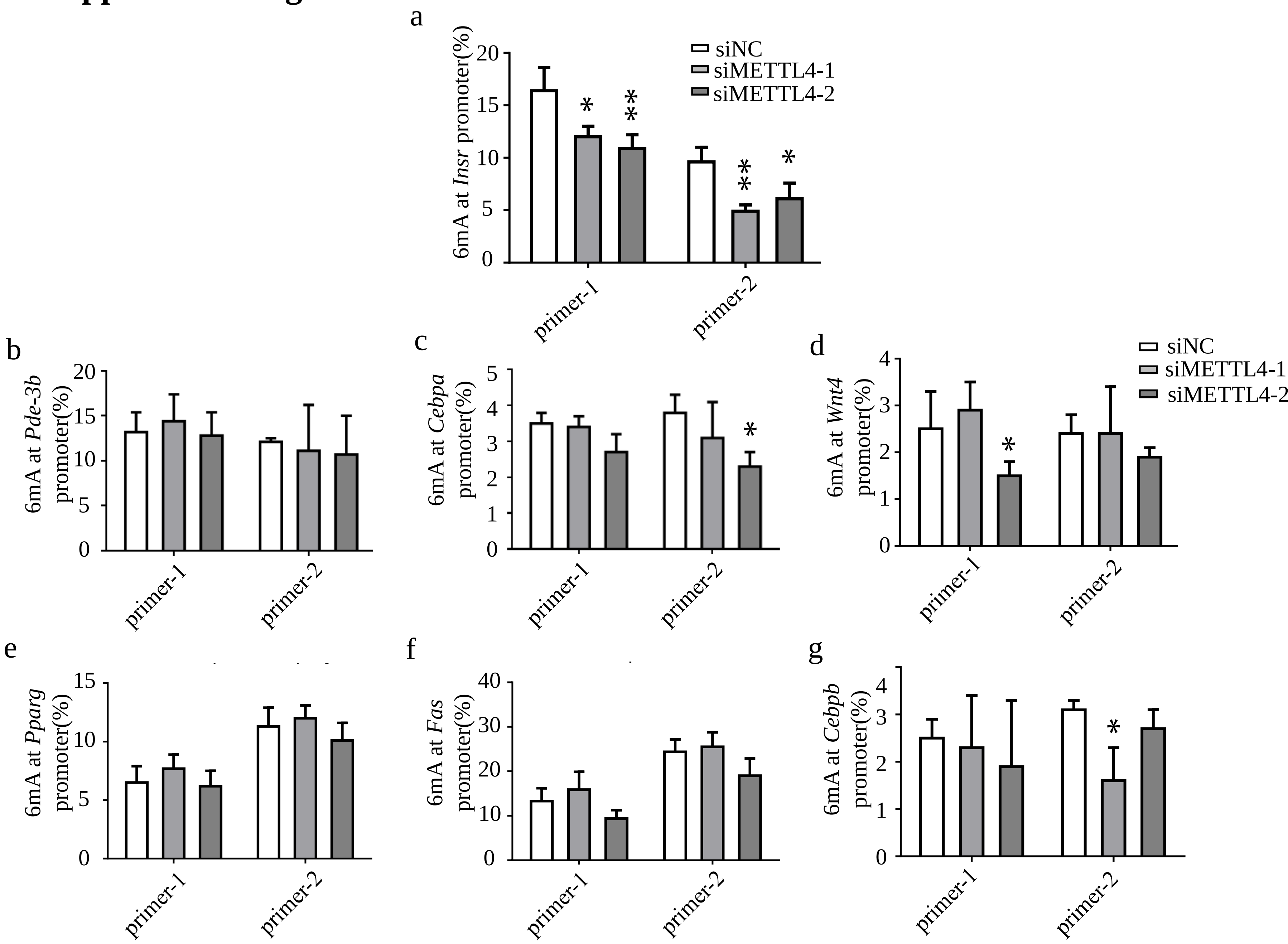

Supplemental Figure S9. Effects of *Mettl4* knockdown on day(+4) of the differentiation.6mA-IP-qPCR was performed to examine the effects of *Mettl4* knockdown on promoter methylation of *Insr, Pde-3b, Cebpa*, *Wnt4*, *Pparg*, *Fas and Cebpb*. (a-g) Promoter 6mA methylation of the genes.

Supplemental Table S1.

ss-oligo1	5'-CATGATACCTTATGGAAAGCATGCTTGTATTTCTT ATGAACCATGATACCTTATGGAAAGCATGCTTGTAT TTCTTATGAACCCGCGCAGGCTGCT
ss-oligo2	5'-ATCGATCGATCGATCGATCG
ds-oligo1	F:5'- CATGATACCTTATGGAAAGCATGCTTGTATTTCTT ATGAACCATGATACCTTATGGAAAGCATGCTTGTAT TTCTTATGAACCCGCGCAGGCTGCT
	R:5'- AGCAGCCTGCGCGGGTTCATAAGAAATACAAGCATG CTTTCCATAAGGTATCATGGTTCATAAGAAATACAAGCAT GCTTTCCATAAGGTATCATG

Supplemental Table S1. Sequence of DNA oligos used in methylation assay in vitro.

Supplemental Table S2.

pde-3B-1	Forward primer: 5'- TTGTCATGCCCTTCTATATGA ACTCAA
	Reverse primer: 5'- CTGTACATAAAATCATCACAGACCACACA
pde-3B-2	Forward primer: 5'- GGGTGTGTGGTCTGTGATGA
	Reverse primer: 5'- TGGTTACCCAAATGTCCTAAATGA
Cebpa-1	Forward primer: 5'- AAAGCAGTCTCCAACCTCCC
	Reverse primer: 5'- ACCGTAGTGCAGACTTGTGT
Cebpa-2	Forward primer: 5'- ACACAAGTCTGCACTACGGT
	Reverse primer: 5'- CTTCCAGCCAACACTAGGGA
Wnt4-1	Forward primer: 5'- GCCAGGCATGGGTTTTTAAGC
	Reverse primer: 5'- GTCTGCTTGGGTTTCTGTTCG
Wnt4-2	Forward primer: 5'- AGGGTTGAATCTGCCTGACG
	Reverse primer: 5'- CAGCGAGGTTTTGAGCGAAC

Pparg-1	Forward primer: 5’- TCTGGTGAGGATGGTTTGTACC
	Reverse primer: 5’- TTGCTCAAGATGCTTCTCCACT
Pparg-2	Forward primer: 5’- GGATGCTAAGAGTGGAGAAGCA
	Reverse primer: 5’- AGGACACCAAAGGCCGTTCT
Fas-1	Forward primer: 5’- CGAGGAGATGAAACCTAGGGC
	Reverse primer: 5’- AGCATCGGTTAATGCTCTTTGA
Fas-2	Forward primer: 5’- TTGTCATGCCCTTCTATATGA ACTCAA
	Reverse primer: 5’- CTGTACATAAAATCATCACAGACCACACA
Cebpb-1	Forward primer: 5’- AGACACAGTGTGGCAGAACC
	Reverse primer: 5’- CTCCACCCTATGTATGGCCC
Cebpb-2	Forward primer: 5’- GGTAGGCGGGCCATACATAG
	Reverse primer: 5’- GGGAAGCAGAACTCTCCAGG
Insr-1	Forward primer: 5’- CTAATCAGTACCCCCAACCC
	Reverse primer: 5’- CCACAGTACAGGTGAATGCC
Insr-2	Forward primer: 5’- ACCTGGAGTTCACTGGCTGA
	Reverse primer: 5’- TGTCCCTTAGGAGCTGTTCAC

Supplemental Table S2. Sequence of primers used in 6mA-IP-qPCR.
